# Supplementary material for: Influence of Chiral Compounds on the Oxygen Evolution Reaction (OER) in the Water Splitting Process
Source: Molecules. 2020 Sep 1;25(17):3988. doi: 10.3390/molecules25173988 (PMC7504774; doi:10.3390/molecules25173988)
Supplement: Supplementary file 1 [file molecules-25-03988-s001.pdf]

molecules

Supplementary Materials

Influence of Chiral Compounds on the Oxygen Evolution Reaction (OER) in the Water Splitting Process

Mirko Gazzotti [1], Andrea Stefani [1], Marco Bonechi [2], Walter Giurlani [2], Massimo Innocenti [2,*] and Claudio Fontanesi [1,*]

[1] Department of Engineering 'Enzo Ferrari', University of Modena and Reggio Emilia, Via Vivarelli 10, 41125 Modena, Italy; gazzotti.mirko@gmail.com (M.G.); 181878@studenti.unimore.it (A.S.);
[2] Department of Chemistry, University of Firenze, via della Lastruccia 3, 50019 Sesto Fiorentino, Firenze, Italy; marco.bonechi@hotmail.it (M.B.); walter.giurlani@unifi.it (W.G.)
* Correspondence: m.innocenti@unifi.it (M.I); claudio.fontanesi@unimore.it (C.F.)

Received: 2 July 2020; Accepted: 27 August 2020; Published: date
Academic Editor: Mark Symes

Abstract: Results are presented concerning the influence on the water splitting process of enantiopure tartaric acid present in bulk solution. Stainless steel and electrodeposited nickel are used as working electrode (WE) surface. The latter is obtained by electrodeposition on the two poles of a magnet. The influence and role played by the chiral compound in solution has been assessed by comparing the current values, in cyclic voltammetry (CV) experiments, recorded in the potential range at which oxygen evolution reaction (OER) occurs. In the case of tartaric acid and nickel WE a spin polarization of about 4% is found. The use of the chiral environment (bulk solution) and ferromagnetic chiral Ni electrode allows for observing the OER at a more favourable potential: about 50 mV (i.e., a cathodic, less positive, shift of the potential at which the oxygen evolution is observed).

Keywords: spin dependent electrochemistry; water splitting; nickel; chirality; OER

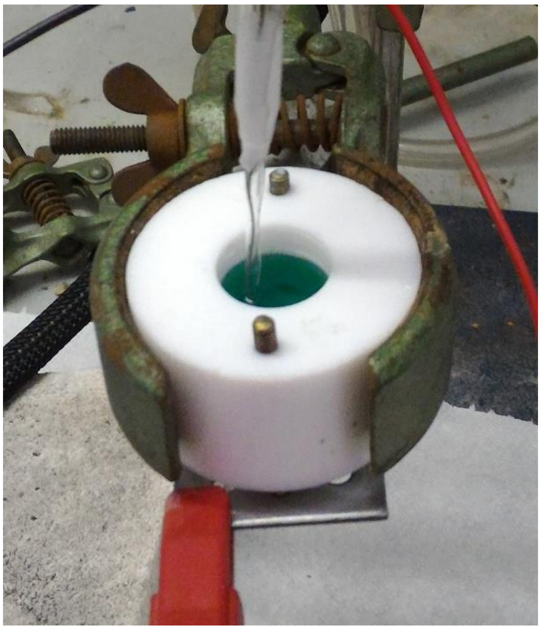

Figure S1. Snapshot of the electrochemical cell set-up.

Table SI. First column: name of the experiment for its unambiguous identification. Second Column: type of electrolyte. Third column: surface used as working electrode. Fourth column: potential at which the oxygen evolution is detected by eye with bubbles formation. Fifth column: stepped sweeps potential vs time function.

Test Name	Electrolyte	Working Electrode	OER [V]	E [V] vs t [s] function
SeS01	KOH 0.1 M	AISI 316L	0.78	
SeS01	TA 0.5mM in KOH 0.1M	AISI 316L	0.75	1
SeS01	TA 5mM in KOH 0.1M	AISI 316L	0.80	
SeS02	KOH 0.1 M	AISI 316L	0.78	2
SeS02	TA 0.5mM in KOH 0.1M	AISI 316L	0.72	
SeS02	TA 5mM in KOH 0.1M	AISI 316L	0.81	1
SeS03	KOH 0.1 M	AISI 316L	0.78	1
SeS03	TA 0.5mM in KOH 0.1M	AISI 316L	0.71	
SeS03	TA 5mM in KOH 0.1M	AISI 316L	0.80	
SeS04	KOH 0.1 M	AISI 316L	0.78	
SeS01	KOH 0.1 M	Ni on North Pole Disk Magnet	0.76	A
SeS02	KOH 0.1 M	Ni on North Pole Disk Magnet	0.76	A
SeS03	KOH 0.1 M	Ni on North Pole Disk Magnet	0.72	E
SeS04	KOH 0.1 M	Ni on North Pole Disk Magnet	0.72	E
SeS05	KOH 0.1 M	Ni on North Pole Disk Magnet	0.71	B
SeS06	KOH 0.1 M	Ni on North Pole Disk Magnet	0.71	B
SeS01	KOH 0.1 M	Ni on South Pole Disk Magnet	0.75	A
SeS02	KOH 0.1 M	Ni on South Pole Disk Magnet	0.75	A
SeS03	KOH 0.1 M	Ni on South Pole Disk Magnet	0.73	E
SeS04	KOH 0.1 M	Ni on South Pole Disk Magnet	0.74	E
SeS05	KOH 0.1 M	Ni on South Pole Disk Magnet	0.72	B
SeS06	KOH 0.1 M	Ni on South Pole Disk Magnet	0.71	B
SeS01	TA 0.5mM in KOH 0.1M	Ni on North Pole Disk Magnet	0.75	A
SeS02	TA 0.5mM in KOH 0.1M	Ni on North Pole Disk Magnet	0.75	A
SeS05	TA 0.5mM in KOH 0.1M	Ni on North Pole Disk Magnet	0.70	B
SeS06	TA 0.5mM in KOH 0.1M	Ni on North Pole Disk Magnet	0.70	B
SeS07	TA 0.5mM in KOH 0.1M	Ni on North Pole Disk Magnet	0.66	C
SeS08	TA 0.5mM in KOH 0.1M	Ni on North Pole Disk Magnet	0.66	C
SeS09	TA 0.5mM in KOH 0.1M	Ni on North Pole Disk Magnet	0.63	D
SeS10	TA 0.5mM in KOH 0.1M	Ni on North Pole Disk Magnet	0.63	D
SeS01	TA 0.5mM in KOH 0.1M	Ni on South Pole Disk Magnet	0.75	A
SeS02	TA 0.5mM in KOH 0.1M	Ni on South Pole Disk Magnet	0.75	A
SeS05	TA 0.5mM in KOH 0.1M	Ni on South Pole Disk Magnet	0.71	B
SeS06	TA 0.5mM in KOH 0.1M	Ni on South Pole Disk Magnet	0.71	B
SeS07	TA 0.5mM in KOH 0.1M	Ni on South Pole Disk Magnet	0.69	C
SeS08	TA 0.5mM in KOH 0.1M	Ni on South Pole Disk Magnet	0.69	C
SeS09	TA 0.5mM in KOH 0.1M	Ni on South Pole Disk Magnet	0.69	
SeS01	KOH 0.1 M	Ni on North Pole Disk Magnet	0.76	A
SeS02	KOH 0.1 M	Ni on North Pole Disk Magnet	0.75	A
SeS03	KOH 0.1 M	Ni on North Pole Disk Magnet	0.71	E
SeS04	KOH 0.1 M	Ni on North Pole Disk Magnet	0.71	E
SeS05	KOH 0.1 M	Ni on North Pole Disk Magnet	0.70	B
SeS06	KOH 0.1 M	Ni on North Pole Disk Magnet	0.70	B
SeS01	KOH 0.1 M	Ni on South Pole Disk Magnet	0.76	A
SeS02	KOH 0.1 M	Ni on South Pole Disk Magnet	0.76	A

SeS03	KOH 0.1 M	Ni on South Pole Disk Magnet	0.76	E
SeS04	KOH 0.1 M	Ni on South Pole Disk Magnet	0.75	E
SeS05	KOH 0.1 M	Ni on South Pole Disk Magnet	0.76	B
SeS06	KOH 0.1 M	Ni on South Pole Disk Magnet	0.78	B
SeS01	TA 0.5mM in KOH 0.1M	Ni on North Pole Disk Magnet	0.75	A
SeS02	TA 0.5mM in KOH 0.1M	Ni on North Pole Disk Magnet	0.75	A
SeS05	TA 0.5mM in KOH 0.1M	Ni on North Pole Disk Magnet	0.70	B
SeS06	TA 0.5mM in KOH 0.1M	Ni on North Pole Disk Magnet	0.70	B
SeS07	TA 0.5mM in KOH 0.1M	Ni on North Pole Disk Magnet	0.67	C
SeS08	TA 0.5mM in KOH 0.1M	Ni on North Pole Disk Magnet	0.67	C
SeS09	TA 0.5mM in KOH 0.1M	Ni on North Pole Disk Magnet	0.63	G
SeS10	TA 0.5mM in KOH 0.1M	Ni on North Pole Disk Magnet	0.64	G
SeS01	TA 0.5mM in KOH 0.1M	Ni on South Pole Disk Magnet	0.76	A
SeS02	TA 0.5mM in KOH 0.1M	Ni on South Pole Disk Magnet	0.76	A
SeS05	TA 0.5mM in KOH 0.1M	Ni on South Pole Disk Magnet	0.73	B
SeS06	TA 0.5mM in KOH 0.1M	Ni on South Pole Disk Magnet	0.73	B
SeS07	TA 0.5mM in KOH 0.1M	Ni on South Pole Disk Magnet	0.70	C
SeS08	TA 0.5mM in KOH 0.1M	Ni on South Pole Disk Magnet	0.70	C
SeS09	TA 0.5mM in KOH 0.1M	Ni on South Pole Disk Magnet		
SeS01	KOH 0.1 M	Ni on North Pole Disk Magnet	0.76	A
SeS01	TA 0.5mM in KOH 0.1M	Ni on North Pole Disk Magnet	0.75	
SeS01	TA 5mM in KOH 0.1M	Ni on North Pole Disk Magnet	0.76	
SeS05	KOH 0.1 M	Ni on North Pole Disk Magnet	0.70	B
SeS05	TA 0.5mM in KOH 0.1M	Ni on North Pole Disk Magnet	0.70	
SeS05	TA 5mM in KOH 0.1M	Ni on North Pole Disk Magnet	0.70	
SeS01	KOH 0.1 M	Ni on South Pole Disk Magnet	0.76	A
SeS01	TA 0.5mM in KOH 0.1M	Ni on South Pole Disk Magnet	0.76	
SeS01	TA 5mM in KOH 0.1M	Ni on South Pole Disk Magnet	0.77	
SeS05	KOH 0.1 M	Ni on South Pole Disk Magnet	0.71	B
SeS05	TA 0.5mM in KOH 0.1M	Ni on South Pole Disk Magnet	0.72	
SeS05	TA 5mM in KOH 0.1M	Ni on South Pole Disk Magnet	0.72	
SeS01	KOH 0.1 M	Ni on North Pole Parallelepiped Magnet	0.75	
SeS02	KOH 0.1 M	Ni on North Pole Parallelepiped Magnet	0.75	
SeS06	KOH 0.1 M	Ni on North Pole Parallelepiped Magnet	0.70	
SeS01	KOH 0.1 M	Ni on South Pole Parallelepiped Magnet	0.75	
SeS05	KOH 0.1 M	Ni on South Pole Parallelepiped Magnet	0.70	
SeS01	TA 0.5mM in KOH 0.1M	Ni on North Pole Parallelepiped Magnet	0.75	
SeS05	TA 0.5mM in KOH 0.1M	Ni on North Pole Parallelepiped Magnet	0.70	
SeS07	TA 0.5mM in KOH 0.1M	Ni on North Pole Parallelepiped Magnet	0.66	
SeS09	TA 0.5mM in KOH 0.1M	Ni on North Pole Parallelepiped Magnet	0.63	
SeS01	TA 0.5mM in KOH 0.1M	Ni on South Pole Parallelepiped Magnet	0.75	
SeS05	TA 0.5mM in KOH 0.1M	Ni on South Pole Parallelepiped Magnet	0.70	
SeS07	TA 0.5mM in KOH 0.1M	Ni on South Pole Parallelepiped Magnet	0.67	
SeS04	TA 0.5mM in KOH 0.1M	AISI 316L	0.71	
SeS04	TA 5mM in KOH 0.1M	AISI 316L	0.81	
SeS05	KOH 0.1 M	AISI 316L	0.77	
SeS05	TA 0.5mM in KOH 0.1M	AISI 316L	0.70	
SeS05	TA 5mM in KOH 0.1M	AISI 316L	0.80	
SeS06	KOH 0.1 M	AISI 316L	0.76	2
SeS06	TA 0.5mM in KOH 0.1M	AISI 316L	0.70	3
SeS06	TA 5mM in KOH 0.1M	AISI 316L	0.76	2

SeS07	KOH 0.1 M	AISI 316L	0.76	
SeS07	TA 0.5mM in KOH 0.1M	AISI 316L	0.76	2
SeS07	TA 5mM in KOH 0.1M	AISI 316L	0.76	
SeS08	KOH 0.1 M	AISI 316L	0.74	
SeS08	TA 0.5mM in KOH 0.1M	AISI 316L	0.76	
SeS08	TA 5mM in KOH 0.1M	AISI 316L	0.74	
SeS09	KOH 0.1 M	AISI 316L	0.73	
SeS09	TA 0.5mM in KOH 0.1M	AISI 316L	0.72	
SeS09	TA 5mM in KOH 0.1M	AISI 316L	0.73	
SeS10	KOH 0.1 M	AISI 316L	0.73	
SeS10	TA 0.5mM in KOH 0.1M	AISI 316L	0.71	
SeS10	TA 5mM in KOH 0.1M	AISI 316L	0.72	3
SeS11	TA 5mM in KOH 0.1M	AISI 316L	0.72	

Table S2. CV of Ni electrodeposited on both side of the magnet B88X0 of K&J Magnet Inc, used as WE. The table presents current peaks and related potential values, for both the electrolytes support tested. All the CVs are made with Ag/AgCl/KClsat as RE and Pt as CE, with a scan rate of 50 mV/s.

	**NORTH POLE**			
	**L-(+)-Tartaric Acid 0.5 mM in KOH 0.1 M**		**KOH 0.1 M**	
Test Name	J Anodic [A]	E Anodic [V]	J Anodic [A]	E Anodic [V]
CV01_Scan1	6.958E-04	0.569	4.987E-04	0.522
CV01_Scan3	6.177E-04	0.554	3.772E-04	0.510
CV01_Scan5	6.055E-04	0.552	3.902E-04	0.513
CV02_Scan1	6.815E-04	0.559	5.185E-04	0.532
CV02_Scan3	6.378E-04	0.552	4.862E-04	0.522
CV02_Scan5	6.412E-04	0.549	5.075E-04	0.522
CV03_Scan1	7.251E-04	0.554	5.884E-04	0.535
CV03_Scan3	6.744E-04	0.547	5.765E-04	0.527
CV03_Scan5	6.784E-04	0.544	5.942E-04	0.527
	SOUTH POLE			
	L-(+)-Tartaric Acid 0.5 mM in KOH 0.1 M		KOH 0.1 M	
Test Name	J Anodic [A]	E Anodic [V]	J Anodic [A]	E Anodic [V]
CV01_Scan1	1.198E-03	0.574	9.909E-04	0.544
CV01_Scan3	1.021E-03	0.552	8.646E-04	0.552
CV01_Scan5	9.998E-04	0.549	8.804E-04	0.522
CV02_Scan1	1.129E-03	0.559	9.518E-04	0.530
CV02_Scan3	1.046E-03	0.549	9.210E-04	0.525
CV02_Scan5	1.061E-03	0.547	9.439E-04	0.525
CV03_Scan1	1.277E-03	0.556	1.031E-03	0.532
CV03_Scan3	1.134E-03	0.547	9.952E-04	0.527
CV03_Scan5	1.137E-03	0.547	1.013E-03	0.527

Table S3. CV of Ni electrodeposited on both side of the magnet B88X0 of K&J Magnet Inc, used as WE. The table presents current peaks and related potential values, for both the electrolytes support tested. All the CVs are made with Ag/AgCl/KCl$_{sat}$ as RE and Pt as CE, with a scan rate of 50 mV/s.

	**NORTH POLE**			
	**L-(+)-Tartaric Acid 0.5 mM in KOH 0.1 M**		**KOH 0.1 M**	
Test Name	J Anodic [A]	E Anodic [V]	J Anodic [A]	E Anodic [V]
CV01_Scan1	6.485E-04	0.554	3.607E-04	0.525
CV01_Scan3	5.655E-04	0.544	3.144E-04	0.513
CV01_Scan5	5.490E-04	0.542	3.336E-04	0.513
CV02_Scan1	6.259E-04	0.549	3.733E-04	0.518
CV02_Scan3	5.740E-04	0.542	3.755E-04	0.515
CV02_Scan5	5.820E-04	0.542	3.915E-04	0.515
CV03_Scan1	6.482E-04	0.547	4.525E-04	0.518
CV03_Scan3	6.046E-04	0.542	4.633E-04	0.518
CV03_Scan5	6.043E-04	0.540	4.735E-04	0.518
	SOUTH POLE			
	L-(+)-Tartaric Acid 0.5 mM in KOH 0.1 M		KOH 0.1 M	
Test Name	J Anodic [A]	E Anodic [V]	J Anodic [A]	E Anodic [V]
CV01_Scan1	7.272E-04	0.570	4.802E-04	0.601

CV01_Scan3	6.479E-04	0.559	4.060E-04	0.537
CV01_Scan5	6.360E-04	0.557	4.263E-04	0.535
CV02_Scan1	7.111E-04	0.566	5.008E-04	0.547
CV02_Scan3	6.580E-04	0.562	4.668E-04	0.537
CV02_Scan5	6.512E-04	0.559	4.792E-04	0.537
CV03_Scan1	6.924E-04	0.564	5.222E-04	0.549
CV03_Scan3	6.598E-04	0.559	5.136E-04	0.542
CV03_Scan5	6.699E-04	0.559	5.261E-04	0.540

Table S4. CV of Ni electrodeposited on both side of the magnet B88X0 of K&J Magnet Inc, used as WE. The table presents current peaks and related potential values, for both the electrolytes support tested. All the CVs are made with Ag/AgCl/KCl_{sat} as RE and Pt as CE, with a scan rate of 50 mV/s.

	**NORTH POLE**			
	**D-(-)-Tartaric Acid 0.5 mM in KOH 0.1 M**		**KOH 0.1 M**	
Test Name	J Anodic [A]	E Anodic [V]	J Anodic [A]	E Anodic [V]
CV01_Scan1	3.850E-04	0.600	2.606E-04	0.600
CV01_Scan3	3.600E-04	0.600	2.306E-04	0.600
CV01_Scan5	3.515E-04	0.600	2.297E-04	0.600
CV02_Scan1	8.200E-04	0.700	5.441E-04	0.684
CV02_Scan3	7.529E-04	0.688	5.270E-04	0.659
CV02_Scan5	7.452E-04	0.686	5.487E-04	0.659
CV03_Scan1	7.761E-04	0.688	5.875E-04	0.664
CV03_Scan3	7.294E-04	0.686	5.481E-04	0.659
CV03_Scan5	7.266E-04	0.688	5.518E-04	0.662
	SOUTH POLE			
	D-(-)-Tartaric Acid 0.5 mM in KOH 0.1 M		KOH 0.1 M	
Test Name	J Anodic [A]	E Anodic [V]	J Anodic [A]	E Anodic [V]
CV01_Scan1	3.852E-04	0.600	2.745E-04	0.600
CV01_Scan3	2.962E-04	0.600	1.772E-04	0.600
CV01_Scan5	2.776E-04	0.600	1.657E-04	0.600
CV02_Scan1	5.728E-04	0.679	3.300E-04	0.675
CV02_Scan3	5.231E-04	0.667	3.100E-04	0.649
CV02_Scan5	5.188E-04	0.664	3.261E-04	0.647
CV03_Scan1	6.137E-04	0.676	3.911E-04	0.657
CV03_Scan3	5.515E-04	0.671	3.600E-04	0.649
CV03_Scan5	5.527E-04	0.671	3.708E-04	0.652

Table S5. CV of Ni electrodeposited on both side of the magnet B88X0 of K&J Magnet Inc, used as WE. The table presents current peaks and related potential values, for both the electrolytes support tested. All the CVs are made with Ag/AgCl/KCl_{sat} as RE and Pt as CE, with a scan rate of 50 mV/s.

	**NORTH POLE**			
	**D-(-)-Tartaric Acid 0.5 mM in KOH 0.1 M**		**KOH 0.1 M**	
Test Name	J Anodic [A]	E Anodic [V]	J Anodic [A]	E Anodic [V]
CV01_Scan1	5.066E-04	0.600	3.719E-04	0.574
CV01_Scan3	4.323E-04	0.600	2.816E-04	0.562
CV01_Scan5	4.153E-04	0.600	2.825E-04	0.564

CV02_Scan1	7.373E-04	0.688	3.315E-04	0.669
CV02_Scan3	6.662E-04	0.674	4.292E-04	0.654
CV02_Scan5	6.560E-04	0.671	4.552E-04	0.654
CV03_Scan1	6.897E-04	0.674	5.014E-04	0.659
CV03_Scan3	6.323E-04	0.671	4.644E-04	0.657
CV03_Scan5	6.299E-04	0.671	4.687E-04	0.657
	SOUTH POLE			
	D-(-)-Tartaric Acid 0.5 mM in KOH 0.1 M		KOH 0.1 M	
Test Name	J Anodic [A]	E Anodic [V]	J Anodic [A]	E Anodic [V]
CV01_Scan1	3.825E-04	0.600	2.703E-04	0.600
CV01_Scan3	3.353E-04	0.600	1.794E-04	0.600
CV01_Scan5	3.201E-04	0.600	1.694E-04	0.600
CV02_Scan1	5.978E-04	0.674	3.315E-04	0.669
CV02_Scan3	5.408E-04	0.659	3.409E-04	0.645
CV02_Scan5	5.377E-04	0.659	3.409E-04	0.645
CV03_Scan1	6.055E-04	0.667	3.849E-04	0.649
CV03_Scan3	5.640E-04	0.664	3.798E-04	0.647
CV03_Scan5	5.768E-04	0.664	4.044E-04	0.649

Table S6. Ratio of anodic current peaks during CVs of Ni electrodeposited on north and south pole of magnet B88X0 of K&J Magnetic, Inc used as WE with solution of 0.5 mM L-(+)-Tartaric Acid in KOH 0.1 M and KOH 0.1 M as electrolyte support. All the CV are run using Ag/AgCl/KCl_{sat} as RE and Pt as CE, with a scan rate of 50 mV/s. Last column shows the result of the sign function of the difference between the two values in the associated row (North – South).

	ANODIC CURRENT RATIO		sgn (J_{ratio} North - J_{ratio} South)
	J_{ratio} North	J_{ratio} South	
Test Name	J_{Tart} / J_{KOH}	J_{Tart} / J_{KOH}	
CV01_Scan1	1.798	1.514	1
CV01_Scan3	1.799	1.596	1
CV01_Scan5	1.646	1.492	1
CV02_Scan1	1.677	1.420	1
CV02_Scan3	1.529	1.410	1
CV02_Scan5	1.487	1.359	1
CV03_Scan1	1.432	1.326	1
CV03_Scan3	1.305	1.285	1
CV03_Scan5	1.276	1.273	1
CV01_Scan1	1.798	1.514	1
CV01_Scan3	1.799	1.596	1
CV01_Scan5	1.646	1.492	1
CV02_Scan1	1.677	1.420	1
CV02_Scan3	1.529	1.410	1
CV02_Scan5	1.487	1.359	1
CV03_Scan1	1.432	1.326	1
CV03_Scan3	1.305	1.285	1
CV03_Scan5	1.276	1.273	1

Table S7. Ratio of anodic current peaks during CV of Ni electrodeposited on north and south pole of magnet B88X0 of K&J Magnetic, Inc used as WE with solution of 0.5 mM D-(-)-Tartaric Acid in KOH 0.1 M and KOH 0.1 M as electrolyte support. All the CV are run using Ag/AgCl/KClsat as RE and Pt as CE, with a scan rate of 50 mV/s. Last column shows the result of the sign function of the difference between the two values in the associated row (North – South).

	ANODIC CURRENT RATIO		sgn (J_{ratio} North - J_{ratio} South)
	J_{ratio} North	J_{ratio} South	
Test Name	J_{Tart} / J_{KOH}	J_{Tart} / J_{KOH}	
CV01_Scan1	1.477	1.403	1
CV01_Scan3	1.561	1.672	-1
CV01_Scan5	1.530	1.675	-1
CV02_Scan1	1.507	1.736	-1
CV02_Scan3	1.429	1.687	-1
CV02_Scan5	1.358	1.591	-1
CV03_Scan1	1.321	1.569	-1
CV03_Scan3	1.331	1.532	-1
CV03_Scan5	1.317	1.491	-1

CV01_Scan1	1.362	1.415	-1
CV01_Scan3	1.535	1.869	-1
CV01_Scan5	1.470	1.890	-1
CV02_Scan1	2.224	1.803	1
CV02_Scan3	1.552	1.586	-1
CV02_Scan5	1.441	1.577	-1
CV03_Scan1	1.376	1.573	-1
CV03_Scan3	1.362	1.485	-1
CV03_Scan5	1.344	1.426	-1

Table S8. Table of the values of spin polarization (SP%) of the current in the measurement made with L-(+)-tartaric acid. The values used to calculate SP% are the ones recorded and reported in 2SI and 3SI for what concerns the current peaks. Table 6SI, instead, reports the "anodic current ratio".

	ANODIC CURRENT RATIO		Spin Polarization Percentage in L-(+)-tartaric acid
	North Pole	South Pole	
Test Name	J_{Tart} / J_{KOH}	J_{Tart} / J_{KOH}	$SP\% = \frac{\left(\frac{J(Tart)}{J(KOH)}\right)_{(North)} - \left(\frac{J(Tart)}{J(KOH)}\right)_{(South)}}{\left(\frac{J(Tart)}{J(KOH)}\right)_{(North)} + \left(\frac{J(Tart)}{J(KOH)}\right)_{(South)}}$
CV01_Scan1	1.798	1.514	8.56%
CV01_Scan3	1.799	1.596	5.98%
CV01_Scan5	1.646	1.492	4.90%
CV02_Scan1	1.677	1.420	8.29%
CV02_Scan3	1.529	1.410	4.05%
CV02_Scan5	1.487	1.359	4.49%
CV03_Scan1	1.432	1.326	3.86%
CV03_Scan3	1.305	1.285	0.78%
CV03_Scan5	1.276	1.273	0.11%

CV01_Scan1	1.798	1.514	8.56%
CV01_Scan3	1.799	1.596	5.98%
CV01_Scan5	1.646	1.492	4.90%
CV02_Scan1	1.677	1.420	8.29%
CV02_Scan3	1.529	1.410	4.05%
CV02_Scan5	1.487	1.359	4.49%
CV03_Scan1	1.432	1.326	3.86%
CV03_Scan3	1.305	1.285	0.78%
CV03_Scan5	1.276	1.273	0.11%

Table 9SI - Table of the values of spin polarization (SP%) of the current in the measurement made with D-(-)-tartaric acid. The values used to calculate SP% are the ones recorded and reported in Table 4SI and Table 5SI for what concerns the current peaks. Table 7SI, instead, reports the "anodic current ratio".

	ANODIC CURRENT RATIO		Spin Polarization Percentage in D-(-)-tartaric acid
	North Pole	South Pole	
Test Name	J_{Tart} / J_{KOH}	J_{Tart} / J_{KOH}	$SP\% = \frac{\left(\frac{J(Tart)}{J(KOH)}\right)_{(South)} - \left(\frac{J(Tart)}{J(KOH)}\right)_{(North)}}{\left(\frac{J(Tart)}{J(KOH)}\right)_{(North)} + \left(\frac{J(Tart)}{J(KOH)}\right)_{(South)}}$
CV01_Scan1	1.362	1.415	1.90%
CV01_Scan3	1.535	1.869	9.81%
CV01_Scan5	1.470	1.890	12.49%
CV02_Scan1	2.224	1.803	-10.45%
CV02_Scan3	1.552	1.586	1.09%
CV02_Scan5	1.441	1.577	4.51%
CV03_Scan1	1.376	1.573	6.70%
CV03_Scan3	1.362	1.485	4.34%
CV03_Scan5	1.344	1.426	2.97%
CV01_Scan1	1.477	1.403	-2.57%
CV01_Scan3	1.561	1.672	3.42%
CV01_Scan5	1.530	1.675	4.53%
CV02_Scan1	1.507	1.736	7.05%
CV02_Scan3	1.429	1.687	8.30%
CV02_Scan5	1.358	1.591	7.89%
CV03_Scan1	1.321	1.569	8.59%
CV03_Scan3	1.331	1.532	7.03%
CV03_Scan5	1.317	1.491	6.19%
